# What helps and hinders midwives in engaging with pregnant women about stopping smoking? A cross-sectional survey of perceived implementation difficulties among midwives in the North East of England

**DOI:** 10.1186/1748-5908-7-36

**Published:** 2012-04-24

**Authors:** Jane Beenstock, Falko F Sniehotta, Martin White, Ruth Bell, Eugene MG Milne, Vera Araujo-Soares

**Affiliations:** 1 Institute of Health and Society, Newcastle University, Newcastle upon Tyne, UK; 2 North East Strategic Health Authority, Newcastle upon Tyne, UK; 3 School of Medicine and Health, Durham University, Durham, UK

## Abstract

**Background:**

Around 5,000 miscarriages and 300 perinatal deaths per year result from maternal smoking in the United Kingdom. In the northeast of England, 22% of women smoke at delivery compared to 14% nationally. Midwives have designated responsibilities to help pregnant women stop smoking. We aimed to assess perceived implementation difficulties regarding midwives’ roles in smoking cessation in pregnancy.

**Methods:**

A self-completed, anonymous survey was sent to all midwives in northeast England (n = 1,358) that explores the theoretical explanations for implementation difficulties of four behaviours recommended in the National Institute for Health and Clinical Excellence (NICE) guidance: (a) asking a pregnant woman about her smoking behaviour, (b) referring to the stop-smoking service, (c) giving advice about smoking behaviour, and (d) using a carbon monoxide monitor. Questions covering Michie *et al.*’s theoretical domain framework (TDF), describing 11 domains of hypothesised behavioural determinants (*i.e.*, ‘knowledge’, ‘skills’, ‘social/professional role/identity’, ‘beliefs about capabilities’, ‘beliefs about consequences’, ‘motivation and goals’, ‘memory’, ‘attention and decision processes’, ‘environmental context and resources’, ‘social influences’, ‘emotion’, and ‘self-regulation/action planning’), were used to describe perceived implementation difficulties, predict self-reported implementation behaviours, and explore relationships with demographic and professional variables.

**Results:**

The overall response rate was 43% (n = 589). The number of questionnaires analysed was 364, following removal of the delivery-unit midwives, who are not directly involved in providing smoking-cessation services. Participants reported few implementation difficulties, high levels of motivation for all four behaviours and identified smoking-cessation work with their role. Midwives were less certain about the consequences of, and the environmental context and resources available for, engaging in this work relative to other TDF domains. All domains were highly correlated. A principal component analysis showed that a single factor (‘propensity to act’), derived from all domains, explained 66% of variance in theoretical domain measures. The ‘propensity to act’ was predictive of the self-reported behaviour ‘Refer all women who smoke……to NHS Stop Smoking Services’ and mediated the relationship between demographic variables, such as midwives’ main place of work, and behaviour.

**Conclusions:**

Our findings advance understanding of what facilitates and inhibits midwives’ guideline implementation behaviours in relation to smoking cessation and will inform the development of current practice and new interventions. Using the TDF as a self-completion questionnaire is innovative, and this study supports previous research that the TDF is an appropriate tool to understand the behaviour of healthcare professionals.

## Background

Smoking during pregnancy is a cause of fetal mortality, low birth weight, and preterm delivery [1] and increases the risk of congenital anomalies [2]. In the United Kingdom, smoking causes an estimated 5,000 miscarriages, 300 perinatal deaths, and 2,200 preterm deliveries per year [1]. In the northeast of England, 22.2% of women were smoking at the time of delivery in 2009/10 compared to a national average of 14.2% [3].

Midwives have a significant role in influencing pregnant women who smoke. In England, recent guidance on smoking in pregnancy from the National Institute for Health and Clinical Excellence (NICE) [4] recommended behaviours specifically for midwives, including using a carbon monoxide breath test, referring all pregnant women who smoke to stop-smoking services, and providing information to women about the risks to the unborn child.

It is important to understand whether and why current guidelines are not fully implemented and what drives the behaviour of health professionals in order to develop interventions to optimise guideline implementation and effectiveness of clinical care. There is currently no evidence about the extent to which guidelines are implemented by midwives in relation to smoking cessation. However, there is evidence that implementation of NICE guidelines varies between organisations, professional groups, and guidance types [5]. A systematic review of qualitative and quantitative studies relating to interventions with pregnant women who smoke identified a number of factors that impeded the implementation of smoking-cessation interventions, such as concern about the potential negative impact on the relationship between the pregnant woman and midwife, and limited knowledge about the use of guidelines or protocols in practice [6]. This latter factor was derived from studies of service providers in Australia, South Africa, and England.

Scientific theories of behaviour and behaviour change might contribute to our understanding of health professionals’ guideline-implementation behaviours by providing hypotheses and possible explanations for behaviour congruent with current evidence that can then be generalised across contexts [7]. For example, many social cognitive theories of behaviour hypothesise that behaviour is a function of an individual’s intention to perform it and that this intention, in turn, is influenced by beliefs about the behaviour, such as the ease, social acceptability, and consequence of performing the behaviour [8]. A systematic review found consistent and strong relationships between intention and clinical behaviours of healthcare professionals [9]. These findings support the applicability of theoretical models of individual behaviour to health professional practice.

One limiting factor in using psychological theory to predict and explain behaviours has been the large number of overlapping psychological, organisational, and motivational theories of behaviour, which make it difficult to select theoretical models or constructs for implementation research. Michie *et al.* reviewed 128 theoretical constructs derived from 33 relevant theories identified as applicable to implementation science. They used an expert consensus approach to group these constructs based on their commonalities into 11 domains [10]. These domains are (1) knowledge; (2) skills; (3) social/professional role and identity; (4) beliefs about capabilities; (5) beliefs about consequences; (6) motivation and goals; (7) memory, attention, and decision processes; (8) environmental context and resources; (9) social influences; (10) emotion; and (11) self-regulation/action planning. While drawing on theories with different traditions, definitions of behaviour, and intended applications, these domains broadly cover the full range of current scientific explanations for human behaviour hypothesised in current theories, without aiming to maintain the explanatory and causal status the individual constructs have in their respective theory. The aggregated nature of the theoretical domain framework (TDF) provides a pragmatic framework for the exploration of behavioural predictors. It has been used to investigate perceived difficulties encountered by health and social care professionals when implementing national guidelines using a focus group approach [11] and clinicians’ behaviour in relation to blood transfusions through semi-structured one-on-one interviews with consultants [12]. Both studies concluded that using the TDF was particularly useful for initially exploring possible explanations for suboptimal implementation behaviours and suggesting the most suitable theories for a further investigation of these behaviours. Moreover, a systematic review of studies predicting clinician’s behaviour using social cognitive theories [13] used the TDF, plus other psychological factors, to categorise the variables used in these studies to predict intention and behaviour of health professionals. A recent study used a survey approach to identify implementation difficulties in delivering tobacco prevention and cessation counselling through dentists and dental hygienists in Finland [14]. This study demonstrated the feasibility of identifying implementation difficulties through a survey based on the TDF and identified issues around environmental context and resources as the main implementation difficulty amongst dental providers. However, the relatively small and heterogeneous sample in the study, limited reliability of the domain survey measures, and the lack of an assessment of actual individual implementation behaviours limits the conclusions that can be drawn from this study.

Our study had two aims. Firstly, we aimed to investigate the perceived implementation difficulties of midwives, working in different roles and locations, in providing smoking-cessation advice to pregnant women who smoke. In line with Michie *et al.*’s TDF [10], we investigated perceptions that were in favour of, and in conflict with, giving advice as specified in the NICE guidance. Secondly, we sought insight into any relationship between the self-reported behaviour of referring women to smoking-cessation services and demographic and professional variables. This work was developed with the purpose of presenting the survey findings to midwives to help them determine what actions would support them in working more effectively with pregnant women who smoke. Results of this initiative will be reported in a separate paper.

## Methods

We conducted a self-reported, anonymous cross-sectional survey.

### Setting and participants

Participants were midwives employed in any of the eight acute National Health Service (NHS) hospital trusts in the northeast region of England, a region with around 30,000 deliveries per year.

### Instrument

The questionnaire was designed to assess the 11 psychological domains identified by Michie *et al.*[10]. Questions were based on the behaviours recommended in the NICE guidance [4]. These are (a) asking a pregnant woman about her smoking behaviour, (b) referring a pregnant woman to the stop-smoking service, (c) giving advice to a pregnant woman about her smoking behaviour, and (d) using a carbon monoxide monitor to assess a woman’s smoking status. These four behaviours were used once in relation to each domain. We used at least three questions relating to each domain to ensure that each domain was accurately assessed. The questionnaire used the evidence statements from a NICE systematic review [6] to inform the topic areas for the questions. In addition, other important behaviours identified as barriers to effective smoking-cessation advice, such as advising women to cut down rather than quit [6], were included. There were 47 questions overall. Questions on midwife behaviours and theoretical domains were randomly ordered in the final version. A free-text section was included at the end of the questionnaire for respondents to add comments on how midwives could best manage pregnant women who smoke or on issues raised by the questionnaire. Responses to each question were assessed on a five-point Likert scale, ranging from ‘strongly agree’ to ‘strongly disagree’. The questionnaire is available in Additional file 1. Table 1 shows the alignment of the evidence statements from the NICE systematic review and the domains. A summary of the constructs relating to each domain is shown in Table 2. We also collected data on age, smoking status, main place of work (*e.g.*, community or delivery suite), employing trust, whether or not respondents had trained as a specialist in smoking cessation, and length of time respondents had been in midwifery practice.

**Table 1:** Evidence statements from the NICE systematic review and the construct domains

**Construct domain**	**Evidence statement from NICE systematic review (12 studies service providers and 11 studies service users)**
Action planning	No evidence statements relevant
Beliefs about capabilities	**Evidence statement 6.** Evidence from four qualitative studies, three surveys, and a study narrative suggests that record-keeping practices and follow-up enquiry may be inconsistent amongst practitioners. Pregnant women smokers and recent mothers differed in their views regarding the frequency with which they should be asked about their smoking. (three studies service users, three studies service providers, and one narrative)
Beliefs about consequences	**Evidence statement 3.** Five qualitative papers describe how the style or way that information/advice is communicated to pregnant women smokers can impact on how the advice or information is received. Concerns regarding advice being construed as nagging or preaching are reported, together with the recommendation that a more caring, empathetic approach may be helpful. (four studies service users, one study service providers)
Environmental context and resources	**Evidence statement 8.** Two qualitative studies, seven surveys, and one narrative provide evidence that staff perceive that lack of time is a significant barrier to the implementation of smoking-cessation interventions. (nine studies service providers and one narrative) **Evidence statement 9.** One qualitative study, six surveys, and narrative from one study suggest that staff perceive that limited resources in the form of either staffing or patient education materials impact on the delivery of interventions. These papers report findings from Australia and the United States, with no UK studies, which may require consideration in terms of applicability to the UK context. (seven studies service providers and one narrative)
Emotion	**Evidence statement 1.** Two qualitative studies and five survey studies provide evidence that not all staff ask all pregnant women about their smoking status during consultations. (three studies service users and four studies service providers) Four studies provide evidence that staff may not ask about smoking status due to concerns regarding damaging the relationship between themselves and a pregnant woman. (two qualitative studies service users, one qualitative study service providers, and one narrative)
Knowledge	**Evidence statement 5.** There is evidence from one qualitative study and two surveys that there is limited knowledge/availability/use of guidelines or protocols in practice. (two studies service providers). There is evidence from one survey that having guidelines/protocols in place may be associated with an increase in the number of smoking interventions offered. (one study service providers) **Evidence statement 10.** Two qualitative studies and seven surveys suggest that staff perceptions regarding the limited effectiveness of interventions may impact on their delivery of services. (nine studies service providers)
Memory, attention, and decision processes	No evidence statements relevant
Motivation and goals	**Evidence statement 2.** Five qualitative studies and three surveys provide evidence that the information and advice currently provided by health professionals is perceived as insufficient or inadequate by some women and by professionals themselves. There is the suggestion that advice could be more detailed and explicit and that professionals find discussion of individual smoking behaviours challenging. (five studies service users and three studies service providers)
Professional role and identity	**Evidence statement 4.** One qualitative study and four surveys provide evidence that there is variance in practice amongst staff in regard to the type of intervention offered during and following a consultation, such as whether a leaflet is offered, whether there is referral on to a specialist programme, or whether ongoing personal support is offered. (two studies service users and three studies service providers) **Evidence statement 11.** Four surveys provide evidence that typical practice in regard to smoking cessation advice and management of care can vary between doctors and midwives. It is reported that general practitioners (GPs) are more likely to advise women to quit smoking completely, whereas midwives are more likely to advise gradual reduction. Also, the evidence suggests that midwives are more likely to refer on to other agencies and record smoking status. GPs may be more likely than midwives to raise the subject of smoking at subsequent consultations. (four studies service providers)
Skills	**Evidence statement 7.** Three qualitative studies, seven surveys, and one narrative report suggest that staff perceive that they have limited skills and knowledge to implement successful smoking-cessation interventions. (one study service users, nine studies service providers, and one narrative)
Social influences	No evidence statements relevant

Evidence statement 12 was not included.

One qualitative study and two narrative reports describe obstacles to pregnant women smokers accessing services as including the length of sessions, difficulty making telephone contact, and a lack of transport or child care.

It is suggested that domiciliary or very local services, the provision of crèche facilities, appointment systems, or telephone counselling could be suitable service delivery options.

**Table 2:** Description of the domains in the context of this survey

**Domain**	**Description in the context of this survey**
**Action planning**	Are there procedures in place to support working with pregnant women who smoke, for example, procedures about how to refer women to the stop-smoking service?
**Beliefs about capabilities**	How difficult or easy is it to support working with pregnant women who smoke? How confident or comfortable do midwives feel about this work?
**Beliefs about consequences**	What do midwives think will happen when they support pregnant women who smoke to stop? What do they see as costs or benefits of this work?
**Emotion**	Do feelings of concern make it easier or harder to support pregnant women who smoke to stop?
**Environmental context and resources**	Are resources available for midwives to support pregnant women who smoke to stop? To what extent do resources help or hinder supporting pregnant women who smoke to stop?
**Knowledge**	What do midwives know about supporting pregnant women who smoke to stop?
**Memory, attention. and decision processes**	Do midwives usually think about smoking cessation when they work with pregnant women? How easy or difficult is it to remember to do it?
**Motivation and goals**	To what extent do midwives want to support pregnant women who smoke to stop? Are there other things that are in conflict with this goal?
**Professional role and identity**	Is this work compatible with professional identity?
**Skills**	Do midwives feel they have the appropriate training to support pregnant women who smoke to stop?
**Social influences**	To what extent do other groups of people influence whether or not midwives support pregnant women who smoke to stop?

### Questionnaire validation

Following the completion of the survey, we undertook a backward validation exercise with five reviewers (health psychologists and applied health scientists) who had a range of knowledge about the TDF. The results of the backwards validation are presented in Additional file 2. In five of the domains, all the questions were correctly matched with the intended domain. Four domains scored 69% or higher. Only half of the questions in the beliefs about consequences domain and 55% in the social influences domain were correctly matched by reviewers. This may explain why in this study there is a lack of distinction between the domains, leading to the high intercorrelations.

### Survey implementation

The paper-based, self-reported survey was undertaken during January and February 2011. All midwives in the region (n = 1,358) were asked to take part via an invitation letter from the head of midwifery in their trust. A reminder letter with another copy of the questionnaire was sent to all midwives, as non-responders could not be identified. They were offered an extension to the closing date after 10 days. On both occasions, a free-post return envelope was included. The questionnaire included no identifying details, and responses were therefore anonymous.

### Data management

Questionnaires were double entered into a Microsoft Excel (Microsoft Corporation, Redmond, WA, USA) spreadsheet for data management and then exported to SPSS 17.0 (SPSS, Inc., Chicago, IL, USA) for analysis. Responses were scored from 1 (strongly disagree) to 5 (strongly agree). Where more than one response was indicated, the variable was coded as missing (for demographic variables) or as least-extreme response (for all other variables). Items worded negatively, such as ‘My attempts to discuss smoking with pregnant women are usually perceived as nagging’, were reverse-coded and had their wording changed for the presentation of the results to the format, ‘My attempts to discuss smoking with pregnant women are not usually perceived as nagging’. Mean scores were calculated for each question. Higher mean scores indicated greater agreement with the statement. An overall mean score was calculated for each of the 11 domains.

Some of the free text responses from midwives indicated that they did not have the use of a carbon monoxide (CO) monitor for measuring smoking status. In addition, in a related audit (unpublished) carried out in parallel with the survey, two trusts stated that not all midwives had access to a CO monitor. Furthermore, the seven questions about CO monitors consistently had high rates of missing responses (15% to 22%). Response rates for all other questions ranged from 94% to 99%. The questions relating to CO monitors were, therefore, excluded from the analysis. Delivery-unit midwives (n = 155) were excluded from the analysis after discussion with midwifery managers, since it was anticipated that they would not be involved in engaging women in discussion about smoking cessation or referring them to stop-smoking services.

### Statistical analysis

Cronbach’s alpha was computed for each domain to assess the internal consistency of the questions in each domain. A linear regression analysis was undertaken to explore which variables were independently associated with the self-reported behaviour ‘I always refer pregnant women who smoke to a stop-smoking service’. This question was selected as the outcome variable because NICE guidance advises midwives to ‘Refer all women who smoke, or have stopped smoking within the last 2 weeks, to NHS Stop Smoking Services’, and referrals are the only behaviour suggested in the NICE guidance that is regularly recorded within each NHS trust. To circumvent possible problems with multi-colinearity in the regression analysis, we conducted a principal component analysis over the highly intercorrelated domain measures to assess whether a more parsimonious structure could describe the TDF data. Moreover, only those demographic variables that had statistically significant correlations with the dependent variable were entered in the regression model.

We tested the hypothesis that the relationship between demographic variables (years practiced as a midwife, age, training in smoking cessation, and main place of work) and self-reported referral behaviour was mediated by the TDF. Mediation was tested using the INDIRECT macro for SPSS [15], which estimated the path coefficients in the mediator model and generated bootstrap confidence intervals (resamples = 5000, bias-corrected and accelerated) for the indirect effects of main place of work on referring to the smoking-cessation service through the proposed mediator variable ‘propensity to act’. Additionally, length of time practiced as a midwife and training as a specialist in smoking cessation were included as covariates in the mediation model.

### Ethics

Ethical approval was granted by a proportionate review subcommittee of Sunderland NHS Research Ethics Committee (10/H0904/75). Research approval was granted by the Research and Development committees in all eight NHS trusts in the region.

## Results

### Characteristics of the respondents

The overall response rate was 43% (n = 589). The number of questionnaires analysed was 364, following removal of the delivery-unit midwives. Table 3 shows the characteristics of participating midwives. Of these respondents, 75% (274) had worked as a midwife for 10 or more years and most (60%, n = 218) worked in the community or an integrated team.

**Table 3:** Demographic characteristics of respondents (n = 364)ª

**Demographic variable**	**n (%)**	
**Years practiced as a midwife**		
10 years or less	89	(24)
More than 10 years	274	(75)
**Age**		
34 years or less	45	(12)
35 to 49 years	200	(55)
50 years or more	116	(32)
**Trained as a smoking-cessation advisor**	57	(16)
**Main place of work**		
In the community or an integrated team	218	(60)
In a fetal medicine unit, day assessment unit, antenatal clinic, inpatient ward, or rotational	146	(40)
**Current smoker**	14	(3)
**Ever smoked**	93	(26)

^a^Some midwives did not answer all questions, so the numbers in each section do not total 364.

### Domain analysis

Table 4 shows descriptive variables and correlations for all domains. Cronbach’s alpha values for the domain measures ranged from 0.61 (beliefs about consequences) to 0.87 (action planning). Nine out of 11 domains had a satisfactory alpha of >0.7. The remaining two were <0.7 but were considered adequate (knowledge = 0.68 and beliefs about consequences = 0.61) given the heterogeneous nature of the TDF.

**Table 4:** Correlations and descriptive variables for all study variables (n = 364)

	**1**	**2**	**3**	**4**	**5**	**6**	**7**	**8**	**9**	**10**	**11**	**12**	**13**	**14**	**15**	**16**	**17**
1 Action planning		.791**	.453**	.434**	.711**	.792**	.475**	.610**	.546**	.778**	.738**	.275**	.259**	–.249**	–.524**	.107*	.041
2 Beliefs about capabilities			.423**	.540**	.639**	.770**	.513**	.659**	.608**	.798**	.646**	.236**	.225**	–.251**	–.425**	.068	–.031
3 Beliefs about consequences				.473**	.533**	.408**	.280**	.480**	.329**	.412**	.440**	.232**	.257**	–.215**	–.262**	–.008	–.018
4 Emotion					.438**	.379**	.409**	.442**	.442**	.464**	.453**	.197**	.178**	–.153**	–.286**	.044	–.058
5 Environmental context and resources						.631**	.476**	.598**	.460**	.672**	.650**	.189**	.246**	–.201**	–.361**	.055	–.047
6 Knowledge							.400**	.566**	.583**	.747**	.610**	.234**	.238**	–.223**	–.404**	.058	.041
7 Memory, attention, and decision processes								.412**	.358**	.443**	.406**	.135*	.143**	–.198**	–.308**	.001	.008
8 Motivation and goals									.625**	.561**	.627**	.194**	.113**	–.123*	–.233**	.055	.016
9 Professional role/identity										.587**	.597**	.160**	.107**	–.140**	–.231**	.049	.048
10 Skills											.668**	.157**	.191**	–.292**	–.477**	.084	–.012
11 Social influences												.228**	.190**	–.186**	–.453**	.047	.041
12 Length of time practiced as a midwife													.647**	–.086	–.251**	.151**	.066
13 Age														–.055	–.251**	.118*	–.076
14 Training as a specialist in smoking cessation															.186**	–.048	.119*
15 Main place of work																–.069	.002
16 Current smoker																	.200*
17 Ever smoked																	
Cronbach’s alpha	0.87	0.81	0.61	0.71	0.70	0.68	0.84	0.78	0.81	0.87	0.80	—	—	—	—	—	—
Mean (SD)	3.83 (0.89)	4.12 (0.69)	3.25 (0.63)	4.07 (0.65)	3.48 (0.79)	4.24 (0.64)	3.59 (0.99)	4.28 (0.57)	4.31 (0.56)	3.77 (0.96)	3.89 (0.68)	—	—	—	—	—	—
Range	1.25 to 5	1 to 5	1.5 to 5	1.5 to 5	1 to 5	2 to 5	1 to 5	2 to 5	2 to 5	1 to 5	1 to 5	—	—	—	—	—	—

SD = standard deviation.

Note: Correlations are reported as Pearson or biserial.

*Correlation is significant at 0.05 level (two-tailed). **Correlation is significant at 0.01 level (two-tailed).

Mean scores for all domains were above the value of 3.00, indicating generally favourable views of participants towards providing smoking-cessation advice to pregnant women who smoke. The two domains with the highest mean scores were ‘professional role and identity’ (4.31, standard deviation [SD] = 0.56) and ‘motivation and goals’ (4.28, SD = 0.57). The two domains with the lowest mean scores were ‘beliefs about consequences’ (3.25, SD = 0.63) and ‘environmental context and resources’ (3.48, SD = 0.79). All domains were highly correlated with each other and significantly but weakly correlated with length of practice as a midwife, age, and training in smoking cessation. Main place of work was moderately and significantly correlated with all domains. There was no correlation with smoking status.

Principal component analysis of the 11 domains identified one component with an eigenvalue of 6.601, accounting for 66% of the variability in TDF scores. This suggests that, in the present study, the psychological domains might best be described as a single factor. For all further analyses, we therefore constructed a grand mean for all 11 domains, which we called ‘propensity to act’, that describes the propensity of midwives to act in relation to pregnant women who smoke.

### Regression analysis

Table 5 shows that professional and demographic variables accounted for 10.6% of the variability in referral behaviour. Adding ‘propensity to act’ to this equation in a second step nearly doubled the predictive utility of the model to an adjusted *R*^2^ = .199 (*p* < .001). ‘Propensity to act’ was the variable that was most strongly independently associated with behaviour, attenuating the beta value for main place of work considerably.

**Table 5:** **Sequential linear regression assessing association of independent variables with referral behaviour**ª

**Independent variables**	***B***	**SE**	***t***	**Adjusted *R***^**2**^
Model 1				.106
Length of time worked as a midwife^b^	.051	.159	.319	
Main place of work^c^	–.843	.141	−6.002**	
Trained in smoking cessation	–.139	.180	–.770	
Model 2				.199
Length of time worked as a midwife^b^	–.100	.152	–.658	
Main place of work^c^	–.422	.149	−2.840*	
Trained in smoking cessation	.082	.174	.470	
Propensity to act	.851	.134	6.373**	

*B* = beta; SE = standard error.

^a^‘I always refer pregnant women who smoke to a stop-smoking service’; ^b^two groups: (1) 10 years or less or (2) more than 10 years; ^c^two groups: (1) in the community or an integrated team or (2) in a fetal medicine unit, day assessment unit, antenatal clinic, inpatient ward or rotational.

*p = .005; **p < .001.

### Mediation analysis

**Figure 1:**
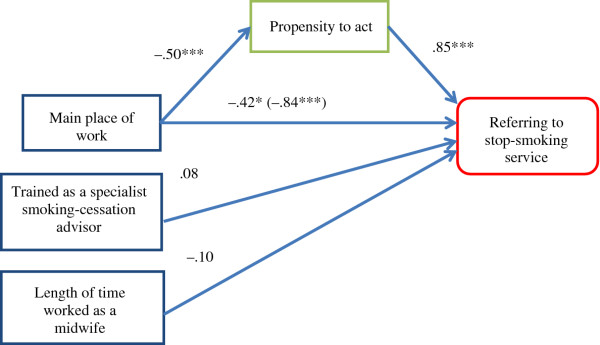
**Mediation model: Path model of the predictive effect of main place of work on the behaviour ‘referring to stop-smoking service’ mediated through ‘propensity to act’ (n = 344)**^**a**^**.**^a^(beta = −.42, 95% confidence interval: –.60, –.27), controlling for length of time practiced as a midwife and trained as a specialist smoking-cessation advisor. *p < .05; ***p < .001.

The hypothesised mediation model in Figure 1 shows that a significant indirect relationship between main place of work and the behaviour ‘refer pregnant women to smoking-cessation service’ acts via ‘propensity to act’ (beta [*B*] = −.42, standard error [SE] = .08 [95% confidence interval: –.60, –.27]). Of the behavioural variance, 20% was accounted for by the hypothesised model. The arrows in Figure 1 reflect the model specification; they do not suggest causality. There was a significant association of main place of work with behaviour (*B* = −.84, SE = .14, *p* < .001; indicates higher probability of referring for midwives working in integrated teams or in the community), if the mediator was not included in the model. The strength of this association decreased when the mediator was included in the model (*B* = −.42, SE = .15, *p* = .05) but remained significant, indicating that there is a significant indirect effect via ‘propensity to act’ and also a direct effect of main place of work on behaviour (partial mediation). Both, the direct effect of main place of work on ‘propensity to act’ (*B* = −.50, SE = .05, *p* < .001) and the effect of ‘propensity to act’ on referring to the smoking-cessation service (*B* = .85, SE = .13, *p* < .001) were significant. Finally, there was no significant effect of control variables on behaviour (trained in smoking-cessation advice: *B* = .08, SE = .17, *p* = .64; length of time practiced as a midwife: *B* = −.10, SE = .15, *p* = .51).

## Discussion

### Summary of main findings

In this survey of a sample of midwives from northeast England using the TDF, respondents mostly displayed favourable views to providing smoking-cessation advice to pregnant women who smoke, had high levels of motivation, and saw this as an integral part of their role. Midwives were less positive about the consequences of their actions in relation to smoking cessation and the environmental context and resources available to them. Using a single-factor solution from a principal component analysis of data items relating to 11 components of the TDF, midwives’ ‘propensity to act’ accounted for the largest proportion of variance and was independently associated with referring pregnant women who smoke to a stop-smoking service. Mediation analysis indicated that main place of work was directly related to referral behaviour, though ‘propensity to act’ had a mediating effect on the relationship.

We were surprised to find that, in this study, all 11 domains were highly correlated and best described as a single homogeneous measure. Amemori *et al.* found substantial relationships between domain measures in their survey of implementation difficulties in tobacco use prevention and cessation counselling with dental providers in Finland, and factor analysis suggested describing the measures along three dimensions (motivation, capability, and opportunity) [14]. These findings reflect that the TDF domains are not a theory. They have been developed to try and encompass a broad range of different theories to arrive at a comprehensive framework that incorporates their main theoretical explanations for behaviour. Due to their descriptive and integrative nature, the domains are not independent constructs, which could explain why in this study the domains were highly correlated with each other. For the present example, ‘propensity to act’ is highly related to place of work. One can argue that midwives in integrated teams and community midwives experience more opportunities to intervene than those in fetal medicine units, day assessment units, antenatal clinics, or inpatient wards. They thus perceive more support, knowledge, and motivation and therefore develop better capabilities over time. This study provides a starting point to understanding implementation difficulties. In order to investigate the specific causal mechanisms of midwives’ implementation of NICE guidance for smoking cessation in pregnancy, further research is needed.

### Strengths and limitations of the methods

Michie *et al.*’s TDF of behaviour change [10] has been used previously in face-to-face interviews to study implementation of a mental health guideline [11] and to understand clinicians’ blood transfusion behaviour [12]. To our knowledge, this study is the first to measure health professionals’ perceived implementation difficulties along the 11 theory domains identified by Michie *et al.*[10] by both self-completion questionnaire and a self-report of implementation behaviour, namely referral of smoking pregnant women to NHS Stop Smoking Services. The questionnaire approach has the advantage of greater reach and thus generalisability, enabling us to assess professional behaviour in a large population over a geographically large administrative area. The questionnaire was based on previous research that has also been used in assessing the implementation of NICE guidance. The behaviours assessed in our questionnaire were drawn from a systematic review that formed the basis of NICE guidance on smoking in pregnancy.

At 43%, the response rate could be considered low, and other cross-sectional surveys of midwives have response rates ranging from 38% [16] to 81% [17]. These surveys were not directly comparable to this one since they were either in different settings (country or area of work) or the questions were focused on other issues. Due to the anonymous nature of the survey, we were unable to quantify the effects of non-response bias, for example, according to midwifery role or age. The survey distribution method in each NHS trust was determined by local midwifery managers and, therefore, subject to some variation. It is possible that not all midwives received copies of the questionnaire. Time limitations associated with funding meant that only one reminder was sent out, which may have limited response further. As usual in survey research, not all midwives completed all the questions, leading to some item response bias [18]. In addition, the questionnaire was perceived as long and repetitive by some respondents. Relatively high mean scores were reported for all domains, indicating that no one disagreed or strongly disagreed with the statements. This could be related to social desirability bias, since it is likely that midwives were aware of national guidance and of their expected professional role in relation to smoking cessation. Two of the 11 domains did not have satisfactory alpha scores, though overall our scores were higher than those reported by Amemori *et al.* In their study, they reported Cronbach alpha scores for nine domains, five of which were <0.6.

Smoking cessation in pregnant women is affected by many interacting factors. These include the socioeconomic status of the pregnant woman, her personal context, and the nature of the relationship that develops between each pregnant woman and the midwife she sees during her pregnancy. This study has focused on only one discrete aspect of the many factors that determine whether or not a pregnant woman who smokes stops. This was a cross-sectional study; therefore, we could only establish association, not causality. The outcome measure was self-reported and, thus, prone to recall or desirability bias. However, it was deemed important at this stage of research to conduct an anonymous survey, as some of the questions on implementation difficulties (*e.g.*, questions about knowledge) might otherwise be subject to social desirability. We therefore decided to rely on self-reports, as data linkage would have undermined the confidence of participants in the anonymous nature of this survey.

### Relationship to existing knowledge

The NICE systematic review [6] reported that some midwives were concerned that they may be perceived as nagging if they gave advice about stopping smoking when pregnant [19]. Our results were consistent with this, as only 19% of respondents agreed that discussing smoking with pregnant women was not usually perceived as nagging.

Whilst Abrahamsson *et al. *[20] reported that Swedish midwives suggested discussing smoking with a pregnant woman was a potential threat to their relationship, our respondents did not seem to identify this as a concern, since 79% disagreed with the statement, ‘Suggesting a woman stops smoking when she is pregnant will make our relationship awkward in the future’. Similarly, NICE evidence statement 7 [6] identifies skills and knowledge as being limiting factors to implementing smoking-cessation interventions, yet in our sample, knowledge was the domain with the second highest score. This could be due to social desirability influencing the responses of our sample, or indeed, could indicate that participants perceived that their training needs were met.

NICE evidence statement 8 [6] highlights time as a barrier to implementing smoking-cessation interventions. In our sample, 39% of respondents disagreed or strongly disagreed that they had enough time to ask women about their smoking behaviour, whilst 41% agreed or strongly agreed they had enough time.

Compared to the study of dental professionals in Finland, this survey had higher internal consistency (α = 0.50 to α = 0.64 for Amemori *et al. *[14], α = 0.61 to α = 0.87 in this study). In both studies, the domain ‘environmental context and resources’ had one of the lowest scores and ‘motivation and goals’ was in the top three highest scores for both studies. However, the results for other domains were different in the two studies. This could reflect the different populations that were studied, in terms of professional expertise, for example, or reflect the ability of the TDF to discriminate between the different perceived implementation difficulties among different healthcare professional groups.

### Implications for clinicians or policy makers

Midwives are a key group of health professionals who can influence pregnant women. It is, therefore, important to understand what helps and hinders their behaviours and what can facilitate them being more effective when they intervene with pregnant women who smoke. Using the TDF has furthered our understanding of what facilitates and inhibits midwives’ behaviour in relation to smoking cessation and offered important pointers for the development of current practice and new interventions. For example, the lower scores for ‘beliefs about consequences’ suggest that midwives may require additional information about the effectiveness of brief interventions in relation to smoking cessation. There were also lower mean scores for the ‘environmental context and resources’ and ‘memory, attention, and decision making’ domains. This suggests that midwives may not have the CO monitors or information leaflets that they require when they are engaging with pregnant women who smoke. The results may also indicate a need for prompts in the process that help remind midwives to ask women about their smoking behaviour. It is also helpful to know that the high scores on the ‘knowledge’ and ‘professional role’ domains indicate midwives are confident that they have a sufficient understanding of what they need to know about smoking in pregnancy and that they consider addressing this topic is part of their work as midwives.

One aspect of the study has been to share the results with midwives in northeast England at a stakeholder workshop. Their interpretation of the results is currently being used to inform actions that they can take to support the work they do with pregnant women who smoke. This work is ongoing and will be reported at a future date.

## Conclusion

This study supports previous research that has found that the use of the TDF offers an appropriate way to understand the behaviour of healthcare professionals. The challenge is to refine the questionnaire so that it can be used either to differentiate more clearly between domains or determine which are the key domains that influence the ‘propensity to act’. Then a shorter (and more acceptable to participants) tool can be developed that could be used more widely as the baseline for assessing the drivers for healthcare professionals’ behaviour, before designing interventions that will change behaviour.

Our findings advance understanding of what facilitates and inhibits midwives’ actions in relation to smoking cessation and will help with the development of current practice and new interventions. Using the TDF as a self-completion questionnaire is innovative, and this study supports previous research that the TDF is an appropriate way to understand the behaviour of healthcare professionals.

## Competing interests

The authors declare that they have no competing interests.

## Authors’ contributions

EM had the idea for the programme of work and, with JB, MW, and RB, developed the initial proposal. JB, VAS, and FFS designed the questionnaire, with input from MW and RB. JB co-ordinated data collection and management. JB, VAS and FFS analysed the data, and all co-authors contributed to interpretation. JB produced the first draft of the manuscript, and all co-authors commented on subsequent drafts and approved the final version for publication.

## Supplementary Material

Additional file 1 Questionnaire “Smoking cessation and pregnant women: what are your views?”. Click here for file

Additional file 2 Results of backward validation exercise. Click here for file
