# Supplementary material for: What helps and hinders midwives in engaging with pregnant women about stopping smoking? A cross-sectional survey of perceived implementation difficulties among midwives in the North East of England
Source: Implement Sci. 2012 Apr 24;7:36. doi: 10.1186/1748-5908-7-36 (PMC3465235; doi:10.1186/1748-5908-7-36)
Supplement: Additional file 1 — Questionnaire “Smoking cessation and pregnant women: what are your views?”. [file 1748-5908-7-36-S1.pdf]

Smoking cessation and pregnant women: what are your views?

Questionnaire

Private and Confidential

Version 3 18.11.10

This questionnaire is for midwives in the North East and is about the work you do with pregnant women who smoke tobacco. The results of the questionnaire will be used in a workshop in March 2011 to which you will be invited. The workshop is being planned by a group which includes two midwives and will aim to identify the best ways to help midwives increase the number of pregnant women who do not smoke when they are pregnant.

There are no right or wrong answers. We are only interested in your views. The questionnaire is both anonymous and confidential. There is no personal identifiable information on the questionnaire and the results will be summarised anonymously from all respondents. It is important we have the views of as many midwives as possible in order that we gain a complete picture. You have received this via your manager, so the research team do not have any personal information about you. You should send this questionnaire directly back to the research team so your manager will not know who has responded and will not see any of the completed questionnaires.

If you have any questions before completing the survey, please contact Jane Beenstock, Specialty Registrar in Public Health (j.beenstock@nhs.net) who is one of the researchers working on this project.

This study has been reviewed by the Sunderland Research Ethics Proportionate Review Sub-Committee.

Completion of this questionnaire is voluntary. It will not be possible to identify you from the survey results. If you complete it we will assume that you consent for the information you provide to be summarised with the other respondents and used to inform the workshop in March 2011. We also hope that the outcome of this work is suitable for publication in peer reviewed and professional journals.

Instructions

Questions answered by placing a tick √ in one of the boxes. For example:

Are you a midwife? Yes [√] No [ ]

If you find a question too difficult to answer or if you do not wish to answer it, please leave it out and move on to the next question.

To what extent do you agree with these statements? Please tick one option for each statement

	Strongly agree	Agree	Neither agree or disagree	Disagree	Strongly disagree
I am aware of the NICE guidance published last year about smoking cessation and pregnant women.	☐	☐	☐	☐	☐
I am actively implementing the NICE guidance published last year about smoking cessation and pregnant women.	☐	☐	☐	☐	☐
Interventions to stop women smoking when they are pregnant are not very effective.	☐	☐	☐	☐	☐
I usually recommend women cut down rather than quit.~	☐	☐	☐	☐	☐
I am confident offering brief advice about smoking cessation to pregnant women.	☐	☐	☐	☐	☐
I am confident offering a brief intervention about smoking cessation to pregnant women.	☐	☐	☐	☐	☐
I am confident in referring pregnant women to the specialist smoking cessation service.	☐	☐	☐	☐	☐
I think it is appropriate for me to follow the NICE recommendations.	☐	☐	☐	☐	☐

	Strongly agree	Agree	Neither agree or disagree	Disagree	Strongly disagree
Following the NICE recommendations could create a problem for my professional autonomy.					
Delivering brief advice about stopping smoking is easy to do.					
Delivering a brief intervention about stopping smoking is easy to do.					
It is important to always about smoking behavior.					
My attempts to discuss stopping smoking are usually perceived as nagging by women.					
The evidence about the benefits of stopping smoking in pregnancy is very strong.					
I want to help women stop smoking when they are pregnant.					
I usually discuss how to stop smoking with women who smoke.					
I usually ask all pregnant women to use the CO monitor.					

	Strongly agree	Agree	Neither agree or disagree	Disagree	Strongly disagree
Suggesting a women stops smoking when she is pregnant will jeopardise my relationship with her.	☐	☐	☐	☐	☐
I have a clear plan of action when I encounter pregnant women who smoke.	☐	☐	☐	☐	☐
I have insufficient time to address smoking cessation with women.	☐	☐	☐	☐	☐
I have insufficient resources to address smoking cessation with women.	☐	☐	☐	☐	☐
Most midwives don't address smoking cessation with pregnant women	☐	☐	☐	☐	☐
Most consultants don't address smoking cessation with pregnant women	☐	☐	☐	☐	☐
Most GPs don't address smoking cessation with pregnant women	☐	☐	☐	☐	☐

Finally, some general information about you……….

Question 1: How many years have you practiced as a midwife?

Less than 2 years ☐

3 to 5 years ☐

5 to 10 years ☐

More than 10 years ☐

Question 2: How old are you?

24 or less ☐

25 – 29 ☐

30 - 34 ☐

35 - 39 ☐

40 - 44 ☐

45 - 49 ☐

50 - 54 ☐

55 - 59 ☐

60 or over ☐

Question 3: Are you a current smoker? Yes ☐ No ☐

Have you ever smoked? Yes ☐ No ☐

If yes, when did you last smoke?;

In the last year ☐

2 to 5 years ago ☐

6 to 10 years ago ☐

More than 10 years ago ☐

Question 4: Which Trust do you work in?

City Hospitals Sunderland NHS Foundation Trust ☐

County Durham and Darlington NHS Foundation Trust ☐

Gateshead Health NHS Foundation Trust ☐

Newcastle Hospitals NHS Foundation Trust ☐

North Tees and Hartlepool NHS Foundation Trust ☐

Northumbria Healthcare NHS Foundation Trust ☐

South Tees Hospitals NHS Foundation Trust ☐

South Tyneside NHS Foundation Trust ☐

If you have any comments or thoughts on how midwives can best manage the problem of smoking in pregnancy, please write them below.

____

____

____

____

____

____

____

This is the end of the questionnaire, thank you very much for taking part. Please return it in the pre-paid envelope provided. If you have any further questions please email Jane Beenstock, j.beenstock@nhs.net.

Thank you!
