# Supplementary material for: What helps and hinders midwives in engaging with pregnant women about stopping smoking? A cross-sectional survey of perceived implementation difficulties among midwives in the North East of England
Source: Implement Sci. 2012 Apr 24;7:36. doi: 10.1186/1748-5908-7-36 (PMC3465235; doi:10.1186/1748-5908-7-36)
Supplement: Additional file 2 — Results of backward validation exercise. [file 1748-5908-7-36-S2.docx]

**Table 2: Results of backward integration exercise**

| **Domain** | **Question number** | **Number of people out of five who correctly identified the intended domain** | **Overall score for the domain** |
| --- | --- | --- | --- |
| **Action planning** | |  | 100% |
|  | 3 | 5 |  |
|  | 16 | 5 |  |
|  | 19 | 5 |  |
|  | 28 | 5 |  |
|  | 43 | 5 |  |
| **Beliefs about capabilities** | |  | 95% |
|  | 20 | 5 |  |
|  | 27 | 5 |  |
|  | 37 | 4 |  |
|  | 46 | 5 |  |
| **Beliefs about consequences** | |  | 50% |
|  | 4 | 3 |  |
|  | 17 | 3 |  |
|  | 25 | 3 |  |
|  | 29 | 1 |  |
| **Emotion** |  |  | 76% |
|  | 1 | 4 |  |
|  | 8 | 4 |  |
|  | 12 | 4 |  |
|  | 26 | 4 |  |
|  | 36 | 3 |  |
| **Environmental context and resources** | | | 80% |
|  | 13 | 2 |  |
|  | 22 | 5 |  |
|  | 30 | 5 |  |
|  | 33 | 5 |  |
|  | 34 | 3 |  |
| **Knowledge** |  |  | 75% |
|  | 7 | 4 |  |
|  | 11 | 1 |  |
|  | 39 | 5 |  |
|  | 41 | 5 |  |
| **Memory, attention, and decision processes** | | | 70% |
|  | 10 | 2 |  |
|  | 18 | 5 |  |
|  | 24 | 5 |  |
|  | 44 | 2 |  |
| **Motivation and goals** | |  | 100% |
|  | 6 | 5 |  |
|  | 15 | 5 |  |
|  | 21 | 5 |  |
| **Professional role** | |  | 100% |
|  | 5 | 5 |  |
|  | 35 | 5 |  |
|  | 38 | 5 |  |
|  | 45 | 5 |  |
| **Skills** |  |  | 95% |
|  | 9 | 4 |  |
|  | 31 | 5 |  |
|  | 40 | 5 |  |
|  | 47 | 5 |  |
| **Social influences** | |  | 64% |
|  | 2 | 2 |  |
|  | 14 | 3 |  |
|  | 23 | 3 |  |
|  | 32 | 4 |  |
|  | 42 | 4 |  |
